# The phenotype of type 1 diabetes in sub-Saharan Africa

**DOI:** 10.3389/fpubh.2023.1014626

**Published:** 2023-01-27

**Authors:** Jean Claude Katte, Timothy J. McDonald, Eugene Sobngwi, Angus G. Jones

**Affiliations:** ^1^Institute of Clinical and Biomedical Sciences, University of Exeter Medical School, Exeter, United Kingdom; ^2^National Obesity Centre and Endocrinology and Metabolic Diseases Unit, Yaounde Central Hospital, Yaoundé, Cameroon; ^3^Academic Department of Clinical Biochemistry, Royal Devon and Exeter NHS Foundation Trust, Exeter, United Kingdom; ^4^Department of Internal Medicine and Specialities, Faculty of Medicine and Biomedical Sciences, University of Yaoundé 1, Yaoundé, Cameroon; ^5^Macleod Diabetes and Endocrine Centre, Royal Devon and Exeter NHS Foundation Trust, Exeter, United Kingdom

**Keywords:** phenotype [MeSH], characteristics, type 1 diabetes, islet autoimmunity, beta-cell, C-peptide, genetics, sub-Saharan Africa

## Abstract

The phenotype of type 1 diabetes in Africa, especially sub-Saharan Africa, is poorly understood. Most previously conducted studies have suggested that type 1 diabetes may have a different phenotype from the classical form of the disease described in western literature. Making an accurate diagnosis of type 1 diabetes in Africa is challenging, given the predominance of atypical diabetes forms and limited resources. The peak age of onset of type 1 diabetes in sub-Saharan Africa seems to occur after 18–20 years. Multiple studies have reported lower rates of islet autoantibodies ranging from 20 to 60% amongst people with type 1 diabetes in African populations, lower than that reported in other populations. Some studies have reported much higher levels of retained endogenous insulin secretion than in type 1 diabetes elsewhere, with lower rates of type 1 diabetes genetic susceptibility and HLA haplotypes. The HLA DR3 appears to be the most predominant HLA haplotype amongst people with type 1 diabetes in sub-Saharan Africa than the HLA DR4 haplotype. Some type 1 diabetes studies in sub-Saharan Africa have been limited by small sample sizes and diverse methods employed. Robust studies close to diabetes onset are sparse. Large prospective studies with well-standardized methodologies in people at or close to diabetes diagnosis in different population groups will be paramount to provide further insight into the phenotype of type 1 diabetes in sub-Saharan Africa.

## Introduction

Type 1 diabetes (T1D), formally referred to as “*juvenile diabetes*” or “*insulin-dependent diabetes mellitus (IDDM)*”, is one of the most commonest diseases of childhood and adolescence, although it can occur at any age, and it is likely that the majority of cases occur in adults (1, 2). The incidence of type 1 diabetes varies considerably from one region of the world to another (3, 4). The classical form of the disease is generally often thought to be less frequent in sub-Saharan Africa (SSA) compared to Europe and North America (5). However, robust epidemiological data on the incidence and prevalence of type 1 diabetes in the African continent are scarce. Estimates from some sub-Saharan African countries have found that the incidence of type 1 diabetes varies from 1.5 to 10.1 per 100 000 depending on the country and age group studied (6). In 2019, the International Diabetes Federation (IDF) estimated that about 25 000 children and adolescents aged <20 years have diabetes in Africa (7). There are suggestions that this number is an underestimation of the true burden of type 1 diabetes in sub-Saharan Africa, given the lack of data from many countries and high disease mortality. It is thought likely that many people with type 1 diabetes may die before a diagnosis is made (8). Therefore, the true economic burden of the condition in Africa is largely unknown.

Atypical young-onset diabetes, including patients with the type 1 diabetes phenotype but who do not appear to have the absolute insulin requirement seen in classical type 1 diabetes, has been reported in African populations (9, 10). Some authors have argued that there may be differences in genetic and autoimmune underpinnings between type 1 diabetes in patients of African and European descent (6, 9, 10), Whether these differences are true remains debatable as most of the studies that have investigated the condition in SSA are limited by small sample size numbers, the use of non-robust type 1 diabetes case definition the use of non-standardized biological assays and the possibility that many reported differences in cross sectional studies (such as high levels of retained insulin secretion) are a result of survival bias (9, 11–13).

Recently, there has been a rising interest in phenotyping type 1 diabetes in African populations. While type 1 diabetes in sub-Saharan Africa is slowly gaining prominence in international research collaborations (11, 14), one key question remains; what is the true phenotype and etiology of type 1 diabetes in sub-Saharan Africa? This has implications for understanding the etio-pathogeny of the condition and providing adequate clinical management strategies. This review aims to provide an up-to-date insight from previously published literature into the characteristics of type 1 diabetes in sub-Saharan Africa, focusing on the clinical description, endogenous insulin secretion, and the contribution of autoimmunity and genetic markers. We also review the context and challenges of type 1 diabetes research and care in sub-Saharan Africa. This understanding is crucial in setting up future research priority areas for type 1 diabetes in sub-Saharan Africa.

## The context and challenges of type 1 diabetes research and care in sub-Saharan Africa

There are several critical challenges to understanding the phenotype and etiology of T1D in SSA. These challenges can be summarized as the absence of robust epidemiological and clinical research on T1D, difficulties in making a correct diagnosis, and suspected mortality attributable to poor insulin access and therapeutic management (9, 13, 15, 16).

The majority of epidemiological and mechanistic research focusing on T1D in SSA dates back to the early 80s and 90s (13). While results were often intriguing, these studies were frequently limited by small sample sizes, limited geographical representation and/or the methodologies employed. Most of the early research interest was fueled by the suggestion that T1D in African populations may differ from the classical form described in western countries, with many studies suggesting links to malnutrition in a subset of those with apparent type 1 diabetes, and ketosis prone type 2 diabetes also described (9, 17). However, this enthusiasm for T1D research diminished in the late 90s and 2000s. This was probably due to the global drive to tackle infectious diseases such as HIV/AIDS, Tuberculosis and Malaria, which were associated with excess mortality in developing countries (18). This targeted focus on infectious diseases meant that funding and research-driven initiatives for non-infectious conditions such as T1D were relegated to the rear of the global health priorities for the region. However, there are a few recent studies from an increasing number of research collaborations between countries in SSA and high-income countries. High-quality longitudinal studies in T1D in SSA are, however, still lacking.

### The problem of case definition and diagnosis of type 1 diabetes in sub-Saharan Africa

The diagnosis of T1D is usually made in symptomatic individuals who present with clinical features suggestive of type 1 diabetes which include younger age, low BMI, ketonuria and presence of autoimmune disease. However, T1D can be difficult to identify clinically, with features overlapping between T1D and other forms of diabetes. As a result, misclassification of diabetes at diagnosis can occur even in countries with high resources and access to classification tests such as islet autoantibodies and C-peptide (19–21). In SSA, the diagnosis of T1D is mainly based on clinical features: classification biomarker tests are rarely available (5). The implications of this are that many people clinically diagnosed with T1D may have other types of diabetes, given the predominance of atypical diabetes presentation in Africa and people of African ancestry.

Atypical presentations of type 2 diabetes (T2D) are known to be predominant in Africa and may further increase the challenge of differentiating between type 1 and 2 diabetes using clinical features alone (9). For example, frequent atypical diabetes forms in SSA include adults with non-obese T2D or those with ketosis-prone diabetes (KPD) (11). Non-obese or lean T2D (BMI <25 kg/m^2^) is common and has been extensively studied in South Asian populations. Studies from Asian populations have demonstrated that non-obese or lean non-autoimmune (‘type 2' diabetes is characterized by younger age and indices of insulin resistance (lower glucose disposal, high insulin levels, higher total abdominal and visceral fat), though beta cell failure remains a critical contributor to the development of diabetes (22–25). While lean T2D has been less comprehensively studied in African populations, a recent study from Uganda found that about one-third of people with adult-onset diabetes had lean non-autoimmune (apparent type 2) diabetes (26). In this study, lean T2D was associated with male sex and beta-cell deficiency without evidence of increased insulin resistance. This means that a significant number of people with low BMI and young age (factors that would support a type 1 diagnosis) in African populations may have non-autoimmune (type 2 diabetes) diabetes.

### The paradox of malnutrition-related diabetes mellitus (MDRD)

Non-autoimmune young-onset diabetes has frequently been described as related to malnutrition in some SSA's impoverished communities. This clinical entity was initially called Malnutrition-Related Diabetes Mellitus (MRDM) (27) and figured in the 1985 World Health Organization (WHO) diabetes classification (28). This was initially described as a diabetes phenotype common in the young (age <30 years), with clinical features of malnutrition, not prone to ketosis, receiving high insulin doses, and living in deprived socioeconomic settings. MRDM is no longer considered a specific diabetes type by the WHO for lack of substantial and consistent data (29). MDRD was further separated into fibro calculous pancreatic diabetes mellitus (FCPD) and protein-deficient or malnutrition-modulated diabetes mellitus. These are atypical forms of diabetes common in lean young males with evidence of chronic clinical malnutrition and could easily be mistaken for type 1 diabetes. These two diseases were described to have similar clinical features with people with FCPD having radiological evidence of pancreatic calcification. There have been numerous reports of FCPD or Tropical Calcific Pancreatitis (TCP) in settings of Africa and Asia (India) that are severely deprived (30, 31). A recent study from a carefully selected group of male adults with low BMI diabetes and a history of malnutrition from India showed they had significantly lower measures of insulin resistance and beta cell function than those with overt type 2 diabetes (32). However, it remains a controversial topic (32, 33).

### Ketosis-prone diabetes is common in African populations and can be mistaken for type 1 diabetes at diagnosis

Ketosis-prone diabetes (KPD) is highly prevalent in Africa and populations of African descent (African-Americans, Afro-Carribeans) but also in some Hispanic and Asian populations (34, 35). People with KPD are often young, middle-aged men who usually present with severe ketosis and high insulin requirements (17). Hence, this group of people with diabetes can easily be mistaken for having T1D. However, they do not have evidence of autoimmunity or type 1 diabetes-related HLA and frequently do not require long-term insulin treatment (17, 36). KPD is also associated with a strong family history of type 2 diabetes with markers of insulin resistance (37–39). Studies have shown that lipid parameters in KPD seem similar to those with T2D in African and Asian populations, although hypertriglyceridemia has been strongly linked to KPD in some Asian populations (40, 41).

In summary, the predominance of atypical presentations of diabetes amongst African populations and limited access to classification tests (islet autoantibodies, C-peptide) in many areas of sub-Saharan Africa mean that robustly diagnosing T1D is challenging, and clinically diagnosed T1D is therefore likely to encompass a range of underlying diabetes pathophysiology. The implications of this are that many phenotype studies may be examining mixed etiology populations, those with and without true autoimmune “type 1” diabetes. This may explain some differences between studies in SSA and T1D cohorts in high-income countries, such as later diabetes onset and lower islet antibody prevalence (5, 6, 42–45).

### High mortality associated with type 1 diabetes in sub-Saharan Africa

T1D in SSA has been associated with very high mortality, especially around the first years of diagnosis (16). This high mortality is often attributed to misdiagnosis and poor insulin access (5, 16, 46). Misdiagnosis of acute diabetes onset, especially in younger children, is common in SSA. It is thought that in many African countries, a substantial proportion of children and young adults with diabetes die before diagnosis or during their first admission from diabetic ketoacidosis (47). Historical evidence suggests that the mortality rate of T1D in Africa after diagnosis may be as high as 80%, similar to the pre-insulin era (16). The reasons for inadequate or poor insulin access in Africa are numerous, ranging from elevated market prices due to the monopoly of few insulin manufacturing industries, challenging regulatory ecosystem for biosimilar “generic” insulins, inadequate procurement and distribution systems to the irrational allocation of health resources within poorly-constructed health systems in Africa (15, 48). Even when insulin treatment is available, its accessibility and proper use is limited due to its high costs and the absence of blood glucose testing devices and accessories needed for adequate control (11, 49).

Early and high type 1 diabetes may significantly affect the phenotype landscape of those with young-onset diabetes. High mortality may alter the phenotype of people with diabetes recruited into cross-sectional studies. Therefore, individuals seen in the multiple cross-sectional studies conducted across SSA may not represent the true population of incident T1D. Hence the phenotype of those who survive (survival effect) may be enriched for those who do not develop absolute insulin deficiency or misdiagnosed individuals with other forms of diabetes.

### The introduction of free insulin donation programmes in sub-Saharan Africa

For the past two decades, sub-Saharan Africa has seen the birth of foreign-funded insulin donation programmes. Examples of such programmes are Life For A Child (LFAC) and Changing Diabetes in Children (CDiC) programmes (50). These programmes tend to enroll and cater for children and young adults <25 years old while providing training and capacity building to health workers toward diagnosing and managing T1D. A crucial observation from these programmes is that a targeted intervention may improve the capacity of health systems in SSA to recognize and diagnose younger-aged children with diabetes even in precarious conditions such as DKA, which were often missed in the past leading to early mortality (51, 52). While the longterm impact of these programmes on the health systems is yet to be completely understood, recent evidence suggests that mortality has improved in countries where these programmes have been introduced (53, 54).

## The clinical characteristics of patients with type 1 diabetes in sub-Saharan Africa

The diagnosis of type 1 diabetes in SSA is primarily or solely based on clinical features. These clinical features include the age of diabetes onset, low or normal BMI, and acute presentation, often with ketoacidosis (55, 56). Table 1 shows summary findings of the clinical characteristics of patients with T1D at or close to diagnosis in SSA. Importantly, in the absence of classification biomarker testing, the clinical features of a population with clinically diagnosed T1D will reflect those features that the clinician considers relevant rather than necessarily the true phenotype of autoimmune etiology diabetes in these populations.

**Table 1:** Summary of studies reporting the clinical features of people with type 1 diabetes in sub-Saharan Africa.

**References**	**Study sample number and population**	**Clinical features reported**	**Notes**
**Studies reporting clinical features in participants at or close to diabetes diagnosis**			
Elamin et al. (57)	A total of 46 children with newly diagnosed IDDM in Sudan.	• Median duration of diabetes: 10 days (range 0-25) • Mean age of onset: 11.6 years • DKA diagnosed: 74% (34/46) • IF-ICA positivity in 58.7% (27/46).	Study was conducted mainly amongst children and adolescents. BMI was not reported. DKA at onset was only clinically diagnosed.
Onyiruika et al. (58)	Retrospective study of 48 children at diabetes diagnosis in Nigeria.	• Mean age of onset: 12.7 ± 2.6 years • DKA diagnosed: 77.1%	DKA diagnosed using a combination of clinical features, hyperglycaemia, acidosis (bicarbonate analysis) and ketonuria. Blood PH was not measured. >50% presented at least 2 weeks after start of symptoms
Asanghanwa et al. (59)	Cross-sectional study of 302 newly diagnosed diabetes individuals and 184 non-diabetic controls in Cameroon	Amongst the 302 diabetes patients, 87 were antibody positive with: • Median duration of diabetes 23 days (range 5-140) • Median age of onset: 30 (19-34 years) • Median BMI 24 (20-28) kg/m^2^ • Ketonuria: 69%	Older age of onset in antibody-defined cases of diabetes. Limitation includes possible misclassification amongst those with positive antibody given higher age of onset and BMI. Islet antibody may be positive in some patients with T2D.
Bahendeka et al. (60)	Cross-sectional study of 85 recently diagnosed T1D children and adolescents in Uganda	• Duration of diabetes < 12 months • Mean age of onset: 15.9 ± 5 years • Mean BMI 18.9 kg/m^2^	Study was conducted primarily amongst children. Peri-pubertal age of onset of diabetes. DKA not assessed
Balcha et al. (61)	Cross-sectional study of 236 recently diagnosed T1D children and adolescents in Ethiopia	• Median duration of diabetes 2.5 months • Median age of onset: 21 years • BMI SD Z score:−1.25 ± 1.15 • Islet antibody positive: 60.6%	Study reveals an older age of onset of diabetes in patients close to diagnosis. DKA not accessed
Mohammed et al. (62)	Cross-sectional study of 99 T1D children and adolescents in Sudan.	• Median age at onset 10.3 years • BMI SD Z score−0.9 ± 2.6 • DKA diagnosed: 55.3% • Islet antibody positive: 69.7%	Pre-pubertal age of onset BMI lower than that of the normal population. DKA was clinically assessed. PH not measured.
Besancon et al. (63)	Cross-sectional study of 130 T1D children and adolescents in Mali aged < 21 years	• Median age at onset 14.6 years • BMI SD Z score:−1.0 ± 1.8 • DKA diagnosed: 44.6%	Study was conducted close diabetes diagnosis. DKA was defined by clinical features with ketonuria
**Studies reporting clinical features in participants with long-duration diabetes**			
Lutale et al. (56)	Cross-sectional study involving 94 T1D and 151 T2D individuals in Tanzania	In participants who were any islet antibody positive (n=40) • Median duration of diabetes: 3.7 years • Median age at onset: 18 years • Mean BMI 20.4 ± 5.4 kg/m^2^ • Family history of diabetes 60%	Post-pubertal age of onset of diabetes Unknown whether family history of diabetes was in first–degree relatives or not.
Gill et al. (42)	Study included 69 insulin treated diabetes individuals and 36 non-insulin treated individuals in Ethiopia.	In the insulin-treated participants with antibody positive (n=18). • Median duration of diabetes 5 years • Median age at onset: 22 years • BMI 18.8 kg/m^2^	Older age of onset in antibody defined patients with T1D. General limitation is the small sample size of the study.
Siraj et al. (64)	Cross-sectional study of 55 T1D and 86 T2D and 46 controls in Ethiopia	In participants with clinically diagnosed T1D • Mean duration of diabetes 6.9 years • Age of onset of diabetes: Not reported • BMI 20.2 kg/m^2^ • Islet antibody positive: 29%	Limitations include, small sample size and age of onset of diabetes not reported.
Govender et al. (65)	Retrospective analysis of 517 individuals with T1D (n=445) and other forms of diabetes in South Africa.	In clinically diagnosed T1D • Mean duration of diabetes 13 ± 9 years • Mean age of onset 15 ± 7 years • BMI 24.4 ± 5.1 kg/m^2^ • DKA in 46.5% (n=207)	Peri-pubertal age of onset of diabetes. Limitation: Retrospective study DKA was defined from medical records
Padoa et al. (66)	Cross-sectional study of 472 (353 black and 119 white) T1D participants in South Africa	In the black T1D participants • Median duration of diabetes 5 years but 8.5 years for white participants • Age at diagnosis: 19 years but 8.5 years for white participants. • BMI: 24 kg/m^2^ and 22.4 kg/m^2^ for white participants	Older age of onset of diabetes amongst black participants compared to white participants.

Ab, Antibody; IDDM, Insulin dependent diabetes mellitus; NIDDM, Non-insulin dependent diabetes mellitus; DKA, Diabetic ketoacidosis; T1D, Type 1 diabetes; T2D, Type 2 diabetes.

### Previous studies show the peak age of onset of T1D is in the second decade of life in sub-Saharan Africa

Traditionally, the peak age of onset of type 1 diabetes has mostly been studied amongst children and adolescents. However, recent evidence suggests that the incidence of T1D in adults may be almost as high as that seen in children and adolescents in developed countries (data from low-and-middle income countries is lacking) (67). Interestingly, a later peak age of onset seems disproportionately reported in the African literature (9). Most studies from SSA have consistently reported that the peak age of onset of T1D occurs about a decade later than the age of diabetes onset observed in people with T1D in Caucasian populations (43, 68). The reason for this observed difference in the peak age of onset of diabetes between patients of African and European ancestry is unknown. High and early mortality among younger patients with T1D in most African countries due to poor medical infrastructure may be a plausible explanation (46, 69). Whether differences in peak age of onset point to heterogeneity in phenotype is unclear. Recent evidence from pancreatic immunohistological studies from developed countries shows that T1D may exist in two distinct endotypes depending on the age of onset of diabetes (70). Findings from these studies have reported that children diagnosed with diabetes <7 years had a hyperimmune insulitis pattern characterized by a lower proportion of residual insulin-containing beta cells, markers of anomalous proinsulin processing, lower peripheral C-peptide levels with higher proinsulin/C-peptide ratios. Contrariwise, children diagnosed with diabetes ≥13 years had a pauci-immune insulitis pattern, with many more beta cells with residual insulin, less signs of anomalous proinsulin processing, higher levels of peripheral C-peptide, and lower proinsulin-to–C-peptide ratios (71–73). The differences observed in the age of diabetes onset in black vs. white South Africans may suggest that a hyperimmune endotype may be common in white vs. pauci-immune endotype being common in black South Africans. Unfortunately, there are no pancreatic immunohistological studies in African populations to assess this hypothesis.

In a recent study in South Africa, Padoa et al. described a bimodal distribution of the age of onset of the disease among the black South African population at 11–15 years and 26–30 years, whereas the peak age of onset was between 0 and 10 years in white South Africans. Also, more than 60% of white South African people with diabetes were diagnosed before 10 years of age (66). A similar pattern has previously been described in black South Africans by Kalk and colleagues (43). Major limitations to these studies are the limited sample sizes, T1D was clinically defined at study onset, only a small proportion of participants were close to diagnosis, and C-peptide was performed only in a small group of participants in the study conducted by Kalk and colleagues. In the study conducted by Padoa and colleagues, the seroprevalence of the GAD65 autoantibody significantly reduced from 73% in those diagnosed at an age < 21 years to 42% in participants diagnosed ≥ 21 years in the black population. This significant reduction was not observed in the white population. Given that atypical diabetes presentations are common in black Africans, the older peak age of onset of diabetes and the reduction of the positivity rate of GAD65 autoantibody may suggest non-autoimmune diabetes in these younger black adults – a finding that has been recently demonstrated in older adults diagnosed with T1D in Europe, where a reduction in islet antibody prevalence with older age in clinically diagnosed T1D appears to reflect misclassification (74, 75). Another recent study in Ethiopia amongst a group of carefully selected patients with young-onset diabetes requiring insulin treatment at diagnosis showed that the median age was 21 years. Only 15.3% were diagnosed at <15 years (61). Though islet autoantibodies were not used to define autoimmune diabetes from onset, the proportion of GAD autoantibody reduced significantly from 80.6% in those <15 years to 43.5% in those >26 years.

In contrast, only a handful of studies in SSA have reported a younger age of onset of T1D, between 10 and 14 years (55, 60, 76), closer to what is described in developed countries. These studies have mainly been conducted in cohorts of children and adolescents with diabetes in insulin donation programs which usually focus on providing care to children under 18 years. Islet autoantibody and C-peptide studies have not been routinely performed in these cohorts. Therefore the proportion of those with true autoimmune T1D is unknown.

### Anthropometric characteristics and family history of diabetes should be considered with caution

Low BMI in young-onset diabetes is usually used as a clinical criterion to identify T1D in SSA. While people with T1D are typically lean, low-to-normal BMI should be considered cautiously since non-obese T2D is common amongst populations of African descent (77). Also, the global increase in the rates of childhood obesity, including in some parts of Africa, may further render the use of BMI challenging in differentiating T1D from T2D. Furthermore, there is increasing evidence that T1D may occur in the presence of obesity (78).

Also, the absence of a family history of diabetes should be avoided as a clinical criterion for T1D. Generally, about 10–20% of individuals with T1D may have a family history of diabetes in a first-degree relative. The risk in first-degree relatives is about 15 times higher than in the general population (79). The heritability risk profile for T1D has been extensively studied in other populations but is utterly unknown in SSA. Few studies from SSA have generally pointed out that about 20–40% of people with T1D have a first-degree relative with diabetes (42, 55, 80). However, the specific type of diabetes was not mentioned.

### Diabetic ketoacidosis (DKA) is a common clinical feature in the presentation of type 1 diabetes

Studies from SSA show that the reported DKA at diabetes diagnosis is highly variable, from 20 and 80% depending on the population studied (55, 58, 80–82). For most of these studies, DKA has not been rigorously defined, with the diagnosis of DKA based on clinical presentation and urine ketones (83). This may explain the high rates in some studies (false positives) when DKA may be absent. Conversely, if DKA is not robustly defined, the false negative rate would be very high. Hence, children with DKA may be misdiagnosed and treated for other conditions, such as severe malaria or meningitis (82, 83). In the few studies where blood ketones confirmed DKA at diagnosis, its prevalence was <40%. This estimate remains substantially high compared to the rate of DKA at diabetes onset in high-income countries (84). In SSA, the time from onset of symptoms to hospital admission can be very long, allowing acidosis to develop in the absence of proper insulin treatment. This may explain why DKA rates are very high in patients with T1D at diagnosis in SSA.

In summary, one may argue that based on current evidence and limitations surrounding case definition in previously conducted studies, and excessively high early type 1 diabetes-associated mortality in African children, it is unclear whether the age of onset of T1D is different in SSA compared to that reported elsewhere. Also, low-to-normal BMI and the absence of a family history of diabetes should not be considered in isolation to make a clinical diagnosis of diabetes in people with young-onset diabetes in SSA. While they are important clinical parameters, they must be considered with other biochemical features to strengthen the robustness of a diagnosis of T1D.

## Type 1 diabetes and autoimmunity in sub-Saharan Africa

Classical type 1 diabetes is a metabolic disease marked by absolute or near-absolute insulin deficiency, usually resulting from autoimmune destruction of the beta cells of the islet of Langerhans (3). The commonest autoantibody markers present in T1D are insulin autoantibodies (IAA), glutamic acid decarboxylase (GAD) autoantibodies, islet antigen 2 (IA-2) autoantibodies and variants of the zinc transporter 8 (ZnT8) autoantibodies (85, 86). Usually, at least one autoantibody is present in over 90% of cases with T1D at the time of diagnosis (75, 86, 87). In clinically suspected T1D, a positive islet antibody will confirm the diagnosis, although the absence of the autoimmune markers does not rule out the presence of the condition (88).

### Studies in sub-Saharan Africa have suggested a lower seroprevalence rate of islet autoantibody in people with type 1 diabetes than elsewhere

Table 2 shows a summary of the seroprevalence of different immunological studies that have been carried out in SSA. These studies have consistently reported lower rates of islet autoantibodies in clinically diagnosed T1D populations than in developed countries (56, 59, 60, 64, 91). This suggests a possible difference in the etiological underpinnings between populations of African and European descent. Racial differences in the prevalence of islet autoantibodies have also been confirmed in immunological studies between black and white individuals living in the African continent. In a more recent analysis of patients with T1D in South Africa, the prevalence of islet antigen 2 (IA2) autoantibodies was 19% (66/352) in black and 41% in white participants (48/118; *P* < 0.001) (66). However, this study did not show any significant difference in GAD (65 kDa) autoantibody positivity between the two ethnicities. The overall median duration of diabetes was 6 years in the study. Furthermore, most immunological studies among people with T1D in SSA have been cross-sectional analyses in people with long-standing diabetes (usually over 3 years).

**Table 2:** Summary of studies reporting the seroprevalence of islet autoantibodies in type 1 diabetes in sub-Saharan Africa.

**Reference**	**Study sample number and population**	**Islet autoantibody assay**	Summary results and notes^***^
**Studies conducted at or close to diabetes diagnosis**			
Mclarty et al. (44)	A total of 122 newly diagnosed black Tanzanian diabetic patients in Dar es Salaam were included in the study; 35 were considered IDDM and 87 NIIDM based on insulin requirement observed in the clinic. The mean age of the different groups was 33 and 46 years.	ICA was measured by indirect immunofluorescence technique (IFL).	The results showed that the seroprevalence of ICA was 8.6% in the IDDM group compared with 6.8% in the NIDDM group. The prevalence of ICA increased to 12% when only participants aged <30 years were considered. ^***^Limitations are: immunofluorescence technique is cumbersome, less specific for islet cell autoantibodies and difficult to standardize. No control group was studied.
Elamin et al. (57)	A total of 46 children with a mean age of 11.6 years with newly diagnosed IDDM were recruited to participate in this study in Sudan. All participants were within 1 month (30 days) from diabetes diagnosis.	Islet-cell antibodies were detected both by the indirect immunofluorescence (IF) and complement fixation (CF) methods.	Sero-prevalence of IF-ICA was 58.7% and CF-ICA was 30.4%. CF-ICA, IF-ICA or both was 63%. ^***^Limitations includes no control group and ICA is known to be non-specific.
Magzoub et al. (89)	A cross-sectional study that involved 96 patients with type 1 diabetes and 86 matched controls in Sudan.	Islet cell antibodies (ICA) was measured in all participants.	The seroprevalence ICA = 41.7 % higher than in controls. ^***^Limitation; Only ICA was measured. ICA is non-specific and may have low sensitivity
Panz et al. (45)	A cross sectional study involving 100 black T1D patients and 80 black T2D, 15 other forms of diabetes and 50 healthy black control participants in South Africa.	Serum GAD autoantibody was measured by radioimmunoassay. The limit of detection was 0.2 U/ml and levels >1 U/ml was considered to be positive.	The prevalence rate of GAD seropositivity was 44% in T1D patients, GAD was positive in 2 patients with T2D and none of the other groups. GAD-positive and GAD-negative participants had similar characteristics (age of onset and duration of diabetes). ^***^The GAD threshold of 1 U/ml may not reflect the optimal cut off for the population and has unclear specificity, although the small control population provides some reassurance.
Rheeder et al. (90)	The study included 43 black and 17 white South Africans with diabetes upon presentation with DKA.	GAD was measured using the ELISA technique. GAD > 50 ng/ml was positive.	Results showed that 33% of the black patients were positive with GAD while 67% of the white patients were positive with GAD. ^***^The GAD positivity rates may have been affected by diabetes duration since white participants were significantly recently diagnosed than the black participants. ^***^Small sample size and lack of population-specific GAD thresholds or control population
Ashanghanwa et al. (59)	A cross-sectional analysis that involved 302 recently diagnosed diabetes patients and 184 non-diabetic controls all aged below 40 years in Cameroon. Of these, 115 were clinically classified as T1D. Also islet antibody positive and negative patients (age-matched) were compared to a patients from the Belgian Diabetes Registry.	GADA, IA-2A, and ZnT8A were measured by liquid phase radiobinding assay. Cut offs were at 99^th^ percentile based on Caucasian populations from the DASP studies.	The seroprevalence of GADA was 24% and 7% in diabetes patients and controls respectively. IA-2A and ZnT8A were very low in both diabetes patients and in controls. In patients who had any antibody positive, GAD titer was not different in the Cameroonian and Belgian populations (82% vs 88%). The seroprevalence of IA-2A and ZnT8A was higher in the Belgian than in the Cameroonian population. ^***^Limitation: The antibody cut-offs were not population-specific.
Bahendeka et al. (60)	Cross-sectional study were 85 recently diagnosed T1D children and adolescents were compared with 79 age-matched controls in Urban Uganda.	GAD, IAA and ZnT8 autoantibodies were measured using ELISA assays and cut offs were set using the manufacturer's specifications.	GAD, IAA and ZnT8 autoantibody positivity rates in the T1D children were 4.9%, 76.8%, 18.3% compared to 29.7%, 60.95, 9.3% in the age-matched controls.
			^***^The data actually shows that islet cell autoantibody positivity rates were as high in T1D patients as in the age-matched controls. This is suggestive of a systematic error (Quality control issues) and illustrates the importance of control population data for defining test specificity. ^***^IAA was measured insulin treated diabetes of up to 1 year duration:insulin treatment may result in false positive results.
Balcha et al. (61)	Cross-sectional analysis of 236 recently diagnosed IDDM patients (<35 years) compared to 200 healthy controls in rural North-West Ethiopia.	Autoantibodies to GAD, IA-2, and ZnT8 were measured by radiobinding assays. The truncated GAD_65_ (96–585) assay was specifically used for the GAD autoantibody due to low control population specificity of the standard assay.	GAD was positive in 55.5% of the T1D and in 2% of the control participants. In T1D participants < 15 years, 86.1% were GAD positive. ^***^These results suggest that most of this group of recently diagnosed T1D individuals may have the similar autoimmune signature as classical T1D in western countries especially those younger than 15 years.
Mohammed et al. (73)	The study involved 99 individuals with clinically diagnosed T1D aged less than 18 years in Sudan.	GADA and IA-2A were measured using commercially available ELISA assays.	GADA and IA-2A positivity rates were 53.5% and 27.2% respectively and 12.1% of the study sample were positive for both, and 69.7% positive for ≥ 1 autoantibody. ^***^High rates of islet antibodies among young and recently diagnosed T1D suggest similar autoimmune aetiopathology for T1D as in Caucasian populations. ^***^Study was conducted in Sudan known to have high Arabic and European genetic admixture.
Besancon et al. (63)	The study involved 132 individuals with young-onset diabetes (age < 21 years) in Mali.	GADA and IA-2A were measured using commercially available ELISA assays.	GADA and IA-2A positivity rates were 58.5% and 21.5% respectively. 11.5% of the participants were positive for both GADA and IA-2A, and 68.5% positive for ≥ 1 autoantibody. ^***^Study shows relatively high rates of overall islet autoantibody prevalence compared to other African studies. ^***^Interesting results as study was conducted in the African Mali population (since Mali has a large Arab population)
**Studies conducted in patients with long-duration diabetes**			
Hawa et al. (91)	Cross-sectional analysis of 101 participants, 47 with T1D, 18 with thyrotoxicosis and, 36 healthy controls in Cameroon.	GADA and IA-2 were tested using radioimmunoassay techniques.	GADA was positive in 34% of the T1D group and 5.6% of the controls. IA-2A was positive in 6.4% of T1D and in 1 control participant. ^***^Findings highlight importance of population-specific cut-offs
Lutale et al. (56)	A total of 245 participants were recruited and they were clinically classified as 94 T1D patients, and 151 T2D patients, at the Muhimbili Hospital in Dar es Salaam, Tanzania.	The islet-cell associated autoantibodies – GADA and IA-2A were measured by radio ligand binding assays.	The seroprevalence of GAD in T1D vs T2D was 21.3% vs 5.3%; IA-2A was 12.8% vs 2%; Any antibody positive 42.6% vs 7.3%; Both antibody positive 8.5% vs 0. ^***^The seropositivity of GAD and IA-2 in the T2D groups reveals the need for population-specific islet autoantibody thresholds. ^***^Double islet autoantibody positivity may be a better immunological T1D identification indicator.
Gill et al. (42)	The study included 69 insulin-treated diabetes individuals were compared to 36 non-insulin treated diabetes individuals attending the diabetes clinic at the Mekelle Hospital in Ethiopia.	GAD antibodies (GADA) and islet antigen 2 (IA-2) antibodies were measured using radiobinding assays.	GADA was positive in 35% of the insulin-treated compared to 14% of the non-insulin treated patients. After re-classification of diabetes types, all patients with “*definite*” T1D having GADA positive, low C-peptide levels and on insulin treatment were different in terms of age of diabetes, and BMI compared to the rest of the groups. ^***^The use of islet antibodies and C-peptide is important in identifying people with T1D ^***^Limitation: Small sample size study
Siraj et al. (64)	Cross sectional study of 187 participants with 55 T1D and 86 T2D and 46 non-diabetic controls in Ethiopia.	ICA was measured using indirect immunofluorescence technique. GADA, IA-2A and IAA were measured using radiobinding assays.	The seroprevalence of GAD in T1D, T2D and controls was 29%, 3.5% and none. The seroprevalence of ICA in the different groups was 21%, 2.7% and none while IAA was 27%, 16% and 2%. IA-2A was absent in all groups. ^***^This results show that GAD continues to be positive in one-third of T1D patients even at a mean of 6 years of disease duration. ^***^The positivity rates of IAA maybe due to insulin treatment in both the T1D and T2D groups (37/86), which may result in false positive results.
Paruk et al. (92)	Cross sectional study among adult 202 T1D individuals attending the T1D clinic at Inkosi Albert Luthuli Hospital in South Africa	Islet-cell associated autoantibody GADA was measured using an ELISA technique.	The seroprevalence of GADA in this population was 63.4%. ^***^High GAD positivity even at long duration of diabetes (mean 10.7 ± 9.1 years).
Padoa et al. (66)	Cross-sectional study of 472 (353 black and 119 white) participants with T1D in South Africa.	GAD65 and IA-2 autoantibodies were measured using an ELISA assays. These were based on the manufacturer's thresolds.	The seroprevalence of IA-2 was 19% in black and 41% in white participants while GAD was 60% in blacks and 66% in the white participants. The seroprevalence of GAD and IA-2 was significantly lower in black participants with age of onset ≥ 21 years. ^***^Data suggests that GAD autoantibody may be long-standing autoimmune marker in this population of T1D given that the median diabetes duration > 5 years.
Bhola et al. (93)	Cross-sectional study among 183 T1D and 49 controls individuals.	ZnT8, GAD65 and IA2 were analyzed using ELISA assays.	Seroprevalence of ZnT8, GAD65, IA2 were 17.5%, 51.3% and 12.8% in T1D patients and 2.0%, 2.1%, 6.5% in control participants. ^***^Participants were initially only clinically diagnosed, and 50% were positive for GAD65 despite having long-duration diabetes.

Ab, Antibody; CV, Coefficient of variation; DASP, Diabetes antibody standardization programme; IDDM, Insulin dependent diabetes mellitus; NIDDM, Non-insulin dependent diabetes mellitus; IFL, Immunofluorescence; DKA, Diabetic ketoacidosis; GADA, Glutamic acid decarboxylase autoantibody; KPD, Ketosis-prone diabetes; PGCP, Post-glucagon C-peptide; RIA, Radioimmunoassay; T1D, Type 1 diabetes; T2D, Type 2 diabetes.

*** was used to mean Take Note.

Some recent immunological studies in insulin-requiring young-onset diabetes individuals have been conducted at or close to diagnosis. For example, in Sudan, a recent study of clinically diagnosed T1D patients <18 years old who were close to diagnosis (<18 months of diabetes duration) showed that GAD and IA-2 autoantibody positivity rates were 53.5 and 27.2%, respectively, with an overall 69.7% positive for at least one autoantibody (62). Also, Balcha and colleagues in Ethiopia showed that the overall seroprevalence of GAD65 autoantibody among newly-diagnosed T1D patients aged <35 years was 55.5% (61). The age-specific seroprevalence of GAD autoantibody was 80.6% (29/36) in those <years, 55.6% (69/124) in the age group 16–25 years, and 43.4% (33/76) in those 26–25 years. IA-2 and ZnT8 positivity rates were generally extremely low irrespective of the age of onset of diabetes. This study enrolled 236 newly-diagnosed patients with insulin-dependent diabetes mellitus (median duration of diabetes was 2.5 months) from a rural setting (61). For the first time, an immunological study done in SSA reported rates of GAD autoantibody as high as that seen in Caucasian populations in those <15 years. It is also worth noting that the GAD assay used in this study was the highly specific truncated GAD65 (96–585) (chosen because of the low specificity of the standard assay in this population described above). These findings underscore that many more studies conducted close to diabetes onset employing robust immunological methods are needed in other parts of the continent to verify these findings in carefully selected cohorts of patients.

These findings are a stark contrast to most of the previous immunological studies conducted in SSA, which have reported low islet autoantibody titers. The differences may come from the timing of the different studies, which were often conducted in groups with varying diabetes duration. Immunological studies among people with T1D close to diagnosis should be encouraged in other African settings. It is important to note that differences may also result from the populations studied. Sudan and Ethiopia are countries located around the horn of Africa with solid historical exchanges with populations in the Middle East, where T1D is very prevalent (94, 95). The Amhara ethnic group from North Ethiopia seem to be a genetically very distinct population group from other black African groups. The relative genetic contribution to T1D susceptibility of this population compared to other black African Bantu and Semi-Bantu populations of SSA to autoimmunity may be different.

In studies where multiple islet autoantibodies have been measured, GAD autoantibody is often strikingly the most commonly detectable autoantibody. The seroprevalence of GAD autoantibody in people with long-standing (>3 years) T1D is estimated at 20–60% (56, 93). Very few studies in SSA have measured IA-2 and ZnT8 autoimmune markers and have reported absent or low rates close to or away from diabetes diagnosis (59, 61, 64, 66). Whether low rates of IA-2 and ZnT8 autoantibodies dispute the relative contribution of these markers to the autoimmune pathogenesis of T1D in SSA is a subject for future research. However, there are suggestions that the differences in the seroprevalence of GADA, IA-2A and ZnT8A observed between Africans, Asians, and Caucasians may arise from differences in background frequency of type 1 diabetes HLA risk alleles (96). For example, HLA DR4 haplotypes are more predominant in Caucasian populations than Africans and Asians. Furthermore, IA-2A and ZnT8A have been shown to be strongly associated with HLA DR4 which may explain their lower prevalence in African populations (96–98). Conversely, HLA DR3 seems to be highly predominant in populations of African ancestries, which may explain the higher rates of GADA in African populations compared to the other islet autoantibodies (59, 66, 96). Therefore, based on current evidence, GAD autoantibody appears to be the single best islet autoantibody to test for in African populations.

In summary, the vast majority of immunological studies conducted among people with T1D or young-onset diabetes have shown very low rates of islet autoimmune markers compared to elsewhere. Two recent studies have demonstrated high islet autoantibody detection rates. Possible reasons for the lower rates of islet autoimmune markers previously reported in multiple studies and their associated limitations are discussed in the subsequent sections.

### Reasons for lower rates of islet autoantibody in people clinically diagnosed with T1D in sub-Saharan Africa

These lower rates of islet autoantibody titers in people with T1D in SSA suggest that non-autoimmune diabetes etiologies may be common in those diagnosed with T1D (9). This may result from the high prevalence of atypical forms of diabetes in African populations (discussed above) that will be extremely challenging to distinguish from autoimmune type 1 diabetes without classification biomarker testing. This may be exacerbated by the low prevalence of ‘true' autoimmune etiology type 1 diabetes in these populations, with studies of confirmed autoimmune type 1 diabetes suggesting autoimmune diabetes is much less frequent than seen in many other regions. Several hypotheses have been put forward to explain lower rates of auto-immune type 1 diabetes in African populations. This includes the widespread practice of infant breastfeeding and “immune priming” from recurrent childhood infections. While prolonged maternal breastfeeding may be protective, there is substantial evidence to show that formula feeding may increase the risk of developing islet-cell autoimmunity (99, 100). Also, recurrent childhood infection is commonplace in SSA and may contribute to immune modulation leading to generally lower rates of autoimmune diseases, including T1D (13, 101). These postulated hypotheses have never been verified in prospective studies in African populations. Other possible mechanisms that could result in lowered islet-cell autoimmunity in those with clinically diagnosed T1D in SSA may be the high prevalence of background intestinal helminth infestation in infants and children (102, 103). Intestinal helminths are well known for modulating their host's immune response. They release immunomodulatory molecules, which are now recognized to interfere with the host's innate and adaptive immune response (104). The impact of such immune molecules on the development of T1D has been extensively studied in animal models but not humans.

Another reason for the lower rates of islet autoantibody in people with a clinician diagnosis of type 1 diabetes in SSA might result from background genetic differences between Africans and other population groups. As discussed in the previous section, there is a great body of evidence suggesting that type 1 diabetes-associated HLA haplotypes DR3 and DR4 are comparatively less predominant in African populations than in Asian, Arabs and European populations (96, 105). A low population background distribution of these HLA haplotypes may influence the islet autoantibody titer levels given the strong association between HLA haplotype and islet autoantibody appearance and titers (86, 87).

### Limitations in many immunological studies conducted amongst people with type 1 diabetes in sub-Saharan Africa

Most immunological studies in sub-Saharan Africa showing lower rates of islet autoimmune markers than those in developed countries have been conducted in small sample numbers. They would usually employ non-standardized methods, reporting positivity thresholds based on cut-offs set by the manufacturer's instructions. Population-specific reference ranges for positive islet autoantibody testing are essential (85, 106). To our knowledge, no study from sub-Saharan Africa has reported population-specific thresholds for islet autoantibodies. Notably, testing 152 controls without diabetes in a recent Ethiopian cohort, using a well-established GAD assay with high specificity in European populations, suggested low specificity with 8.5% of those without diabetes testing positive. This indicates that population-specific thresholds may be particularly important for the robust definition of autoimmune diabetes in African populations.

There is also limited available data from prospective studies on the impact of duration of diabetes on the kinetics of these islet-cell autoimmune markers.

### Characteristics of people with islet autoimmune positive type 1 diabetes in SSA

Numerous studies from SSA show that C-peptide levels are lower in people with T1D who are positive for islet autoantibodies compared to autoantibody negative (42, 59, 61, 90). Other characteristics of islet autoantibody-negative T1D show they are older-aged and have higher BMI (59, 93). This strongly suggests the presence of alternative etiologies of diabetes amongst those with islet autoantibody-negative diabetes.

## Beta-cell function and endogenous insulin secretion in type 1 diabetes patients in sub-Saharan Africa

Type 1 diabetes usually results in near absolute loss of insulin secretion due to the autoimmune destruction of the beta-cells of the islet of Langerhans of the pancreas. Idiopathic loss of endogenous insulin secretion is also defined as type 1 diabetes in some international definitions (3, 107). The loss of endogenous insulin secretion drives the treatment requirements of T1D. Endogenous insulin secretion in those treated with insulin can be assessed using C-peptide, a peptide secreted in equimolar concentrations with insulin (108). Low levels (<200 pmol/L in blood or urine after stimulation) confirm the treatment requirement of type 1 diabetes and are seen in the vast majority of those with young onset type 1 diabetes with a duration of diabetes over 3 years in other populations (108).

### C-peptide levels are low in Type 1 diabetes in sub-Saharan Africa

Table 2 shows the list of studies that have reported on assessing endogenous insulin secretion in SSA. A general limitation applicable to the vast majority of studies is the use of clinically diagnosed T1D, without islet autoantibody confirmation. This means that retained insulin secretion may reflect the classification challenges discussed above rather than a specific phenotype of African autoimmune type 1 diabetes. Studies conducted at or close to diagnosis have generally shown low but detectable C-peptide levels, substantially lower than comparable populations of other diabetes types were available (42, 59, 60, 90, 109). Long-duration studies suggest much higher levels of retained endogenous insulin secretion than in other type 1 diabetes populations. Of note, studies comparing islet antibody-positive and negative participants have consistently shown higher C-peptide levels in antibody-negative individuals. This may indicate the prevalence of other diabetes subtypes in those without islet antibodies, which is also supported by lower type 1 diabetes HLA susceptibility in this group in the recent study by Balcha and colleagues (61).

### Clinical reports suggesting retained endogenous insulin secretion in SSA

Some clinical observations and studies have reported the existence of groups of people with “type 1 diabetes” like phenotype who are insulin-requiring but can interrupt their routine insulin treatment without developing severe metabolic complications (9, 65, 110). This has suggested that some patients with T1D in SSA may have substantial levels of preserved endogenous insulin secretion. However, studies of C-peptide in long-duration clinically diagnosed T1D (Summarized in Table 3) suggest that this will be a modest proportion of patients. In other populations, routine C-peptide testing has led to treatment change and successful insulin withdrawal in a subset of those with clinically diagnosed T1D (20) and is likely to be cost-effective. A similar strategy may have utility in African populations where clinical classification is the mainstay of diagnosis, with the cost of C-peptide testing similar to or less than the cost of a single insulin vial.

**Table 3:** Summary of studies reporting the C-peptide levels and the assessment of endogenous insulin secretion in type 1 diabetes in sub-Saharan Africa.

**Reference**	**Study sample number and population**	**C-peptide test**	Summary results and notes^***^
**Studies at or close to diagnosis of diabetes**			
Elamin et al. (57)	A total of 46 children with a mean age of 11.6 years with newly diagnosed IDDM in Sudan. Duration of diabetes < 30 days.	Fasting C-peptide. Post stimulation C-peptide	About 82.2% of patients had evidence of residual pancreatic beta cell function with stimulated C-peptide at 0.40 nmol/l and 0.53 nmol/l in ICA-positive and ICA-negative children, respectively. ^***^These were newly diagnosed patients, so high C-peptide levels are expected within the first few years of diabetes diagnosis.
Panz et al. (45)	A cross sectional study involving 100 black T1D individuals (age at onset < 35 years, body mass index (BMI) < 27 kg/m^2^ and insulin dependent within 1 year of presentation) in South Africa.	Random C-peptide levels	The average C-peptide level was significantly lower in T1D patients than T2D patients; 0.50 ± 0.03 nmol/l vs 1.13 ± 0.05 nmol/l. The average duration of diabetes was similar between both groups; 3.4 ± 0.4 years vs 5.3 ± 0.6 years.
Bahendeka et al. (60)	Cross-sectional study were 85 recently diagnosed T1D children and adolescents were compared with 79 age-matched controls in Urban Uganda.	Fasting C-peptide was measured. Lower limit of detection was 0.2 nmol/l and was considered low C-peptide levels.	Results showed that the mean C-peptide levels were 0.78 ± 1.26 nmol/l and 1.49 ± 1.49 nmol/l in the T1D and controls, respectively. Half (50%) of the T1D patients had C-peptide ≥0.2 nmol/l. ^***^Recently diagnosed T1D patients may have high levels of residual endogenous insulin secretion.
Balcha et al. (61)	Analysis of 236 recently diagnosed IDDM patients (< 35 years) compared to 200 healthy controls in rural North-West Ethiopia.	Non-fasting C-peptide. Lower limit of detection at 0.0033 nmol/l.	C-peptide was generally low but detectable in the T1D patients, 0.27 (0.11-0.47) nmol/l. GADA-negative patients had a higher C-peptide level than GADA-positive T1D patients; 0.10 nmol/l vs 0.07 nmol/l, p=0.015. ^***^Specific thresholds were not examined in this study. All T1D patients were recently diagnosed, although they generally had low C-peptide levels, as expected in a T1D population.
Mohammed et al. (73)	The study involved 99 individuals with clinically diagnosed T1D aged less than 18 years who were recently diagnosed (< 18 months) in Sudan.	Fasting C-peptide	The mean fasting C-peptide levels was 0.22 ± 0.25 nmol/l and 68.7% had C-peptide < 0.26 nmol/l. ^***^C-peptide levels were generally low consistent with type 1 diabetes although about one-third of the participants had C-peptide levels >0.26 nmol/L. The short duration of diabetes may explain the C-peptide levels in this study.
Besancon et al. (63)	The study involved 132 individuals with young-onset diabetes (age < 21 years) in Mali.	Fasting C-peptide	The mean fasting C-peptide level was 1.2 ± 0.9 nmol/l and 12.3% of the participants had C-peptide < 0.26 nmol/l. ^***^More than half of the participants had high C-peptide levels suggesting significant endogenous insulin secretion (C-peptide was measured 1 week from diabetes diagnosis).
**Studies in long-standing diabetes**			
Siraj et al. (109)	The study included 56 T1D, 97 T2D and 50 non-diabetic controls in Urban Ethiopia.	Fasting C-peptide. Post-stimulation C-peptide.	Results showed that 16% T1D, 93% T2D and 96% non-diabetic controls had C-peptide ≥0.2 nmol/l on the fasting sample. PGCP was measured in 22 T1D and 14% had PGCP levels ≥0.32 nmol/l showing some form of endogenous insulin secretion. ^***^The different diabetes study groups were based on WHO diabetes classification (1980s); therefore a higher chance of misclassification within the diabetes groups.
Lutale et al. (56)	A study of 245 patients, 94 T1D patients and 151 T2D patients, at the Muhimbili Hospital in Dar es Salaam, Tanzania.	Random C-peptide. C-peptide negative and C-peptide positive based on fasting and glucose stimulated C-peptide levels (fasting C-peptide levels ≥0.3 nmol/l or glucose-stimulated C-peptide levels ≥0.6 nmol/l).	Findings showed that 7.6% (7/94) T1D and 63.6% (96/151) T2D had positive C-peptide levels. ^***^Low C-peptide levels was common in those (92.4%) labeled T1D (median duration of diabetes of 17 years).
Gill et al. (42)	The study included 69 insulin-treated diabetes patients and 36 non-insulin treated diabetes patients attending the diabetes clinic at the Mekelle Hospital in Ethiopia.	Fasting C-peptide. C-peptide negative was considered at < 183 pmol/l.	The median C-peptide levels were 477 (286-845) in the insulin-treated group compared to 788 (586-1136) in the non-insulin-treated group. In the insulin-treated group, 61% were C-peptide negative, meaning 39% had detectable C-peptide levels. ^***^The lower limit of detection of < 183 pmol/l is close to the clinically relevant threshold of < 200pmol/l to define severe insulin deficiency. About 40% of insulin-treated patients had C-peptide levels >183 pmol/l. ^***^The data also suggest some initial misclassification given that when low peptide and GAD positivity were combined, only 18 participants who were insulin treated fitted the T1D criteria.
Siraj et al. (64)	Cross sectional study of 187 participants with 55 T1D and 86 T2D and 46 non-diabetic controls in Ethiopia. The mean disease duration for T1D participants was 6 years.	Fasting C-peptide	Basal C-peptide levels were lower in patients identified as having T1D (0.13 ± 0.04 nmol/l) compared to T2D patients (0.67 ± 0.04 nmol/l). ^***^The data did not show the C-peptide levels in those identified as having T1D stratified by their Ab status.
Govender et al. (111)	A retrospective analysis that included 517 patients of whom 445 (86.1%) were diagnosed with T1D, 27 (5.2%) with T2D, 27 with KPD (5.2%) and 18 (3.5%) with other forms of diabetes in South Africa	Fasting C-peptide. Post stimulation C-peptide	Basal and stimulated C-peptide levels were significantly lower in T1D patients compared to the T2D and KPD patients. ^***^Limitation was that data was retrospectively collected.

Ab, Antibody; CV, Coefficient of variation; IDDM, Insulin dependent diabetes mellitus; DKA, Diabetic ketoacidosis; GADA, Glutamic acid decarboxylase autoantibody; KPD, Ketosis-prone diabetes; PGCP, Post-glucagon C-peptide; RIA, Radioimmunoassay; T1D, Type 1 diabetes; T2D, Type 2 diabetes; UCPCR, Urinary C-peptide creatinine ratio.

*** was used to mean Take Note.

## Genetics of type 1 diabetes in sub-Saharan Africa

Genetic factors are known to contribute to the pathogenesis of T1D, and approximately 95% of people with the disease have T1D associated polymorphisms of class II Human Leucocyte Antigen (HLA) genes (88, 112). These HLA genes provide the greatest contribution to overall genetic susceptibility. Type 1 diabetes is known to be strongly associated with HLA-DR3-DQ2 and HLA-DR4-DQ8 haplotypes (87). Apart from these HLA genes, about 60 loci have been associated with susceptibility to T1D through genome-wide association studies (113).

### HLA DR3 has the highest genetic susceptibility for type 1 diabetes patients in sub-Saharan Africa

Table 4 shows a summary of the studies that have been conducted in SSA examining the association between HLA genes and T1D. These studies generally report that the commonly reported HLA genes associated with T1D elsewhere are also found in people with T1D in SSA. In some early studies, Magzoub and colleagues in 1991 showed that the HLA DQB1^*^0201 ad DQA1^*^0301 were strongly associated with IDDM patients in Sudan compared to ethnically-matched non-diabetic controls (115). Several small-scale studies have further described the association between HLA DR3 genes and type 1 diabetes in West and East African populations (115–118). When compared to DR3 genes, DR4 genes seem to be less frequent. Also, HLA genes known to confer protection from T1D have been reported in SSA. In a recent study in North West Ethiopia, GAD-negative diabetes patients had the HLA-DRB1^*^04, including the HLA-DRB1^*^15 alleles (61), and the HLA-DRB1^*^15 allele, which has also been shown in other populations to confer one of the strongest protection for type 1 diabetes (121).

**Table 4:** Studies reporting HLA and genotype of type 1 diabetes in sub-Saharan Africa.

**Reference**	**Study sample number and population**	**Summary results**	**Notes**
McDonald et al. (114)	Cross-sectional analysis of 19 T1D, 50 non-diabetic controls from the Yoruba tribe in Nigeria.	Ten (52.6%) of 19 T1D patients had DR3 positivity compared to 15/50 (30%) controls. Only 2 T1D (11%) and 2 control (4%) participants were DR4 positive. The frequency of DR2 in the T1D patients was low (4/19 vs 23/50).	Major limitation includes the very small sample size and the low-resolution HLA typing method used
Magzoub et al. (115)	Study conducted in 72 IDDM and 59 control participants in Central Sudan.	HLA DQB1*0201 (DQw2) showed a strong positive association with IDDM compared to controls. The DQA1*0301 linked to the DR4 was also positively associated with IDDM.	DR3/DR4 HLA haplotypes are associated with IDDM. The small sample size is a limitation
Garcia-Pacheco et al. (116)	Study conducted in 40 IDDM and 82 controls in a black African population in Zimbabwe.	HLA-DR3 genes; DRB1*0301, DQB1*0201 and DQB1*0302 and DQA1*0301 and DQA1*0501 were significantly increased in the IDDM compared to the control participants. HLA-DR4 gene DRB1*0405 was also significantly higher in the IDDM compared to the control participants.	HLA-DR3 & DR4 genes are associated with T1D
Chuaffert et al. (117)	Cross-sectional analysis of 55 IDDM and 118 non-diabetic controls in Senegal.	HLA DQB1 alleles (0201 and 0302) were positively associated with diabetes. The DQB1*0501 (Val57) allele was less frequent in IDDM subjects than in control subjects.	DQB1*0501 may be protective
Cisse et al. (118)	Cross-sectional analysis of 93 IDDM and 115 control participants in Senegal.	Three main HLA loci were associated with IDDM compared to controls in this study. DQA1*0301 (p < 10^−9^, OR 5.21), DQB1*0201 (p < 10^−7^ OR=3.55) and DQB1*0302 (p < 10^−3^ OR=3.20).	Common DR3 HLA genes are associated with T1D
Mbanya et al. (119)	Study conducted in 10 T1D patients with negative C-peptide and 90 non-diabetic controls in Cameroon.	HLA loci were more frequent in T1D patients compared to controls: DRB1*03, DRB1*1301, DQA1*0301, DQB1*0201. The DRB1*15 (43% vs 0) and DQA1*01 (79% vs 0) frequency were higher in healthy controls compared to the T1D patients.	This study is hugely limited by the small sample size
Ashanghanwa et al. (59)	A cross-sectional analysis that involved 302 recently diagnosed diabetes patients and 184 non-diabetic controls in Cameroon. Findings were compared to patients from the Belgian Diabetes Registry.	Genetic analyses revealed that the high-risk genotype *HLA DQ2/DQ8* was completely absent in the Cameroonian cohort but present in 23% of the islet antibody positive Belgian patients. The frequency of *DQ8/nonDQ2* was also higher in Belgian patients. Cameroonian islet antibody positive patients showed a higher prevalence of *nonDQ8/nonDQ2*.	High risk HLA DQ2/DQ8 haplotype seems not to be present in this population although this may be due to linkage disequilibrium as well.
Fagbemi et al. (120)	A pilot study with HLA typing in 51 T1D and 51 healthy controls using the PCR-SSP method in Benin.	T1D was significantly associated with DR3, DQA1*05:01, DQB1*02:01, and DR3-DR4. No significant associations were observed with DR4, DQB1*03:02, and DQB1*06:02 genes.	DR4 haplotype did not show any association with T1D. The study is limited by its small sample size.
Bahendeka et al. (60)	A cross-sectional analysis of 85 recently diagnosed T1D children and adolescents compared to 79 age-matched controls in Urban Uganda.	The results revealed that only the DQB1*02 was significantly associated with T1D, unadjusted odds ratio (UOR) 4.2 (95% CI 1.4–12.7) (p=0.01). The DRB1*04 also showed a higher allelic frequency in T1D patients compared to the controls.	Contribution of both HLA-DR3 and HLA-DR4 genes to T1D. Limitation include the low genotyping resolution from the HLA typing method employed.
Balcha et al. (61)	Cross-sectional analysis of 236 recently diagnosed T1D patients (< 35 years) compared to 200 healthy controls in rural North-West Ethiopia.	HLA-DRB1*0301 and HLA-DRB1*04 genotypes were associated with T1D in this population compared to controls. The protective haplotype HLA-DRB1^*^15 was more common in controls (25/152, 16.4%; OR 0.20) than in T1D patients (7/188, 3.7%). SNPs within the HLA region; *HLA-DQB1* locus (rs9273363) and *HLA-DQB1* region (rs1063355) showed positive signals. T1D GRS was significantly higher in diabetes participants compared to controls, 0.189 ± 0.064 vs 0.154 ± 0.067.	HLA DR3/DR4 are associated with T1D. HLADRB1*15 may be protective. ^***^ One of the first to use a GRS approach in an African setting and seems to demonstrate that GRS may be useful although this was based on SNPs generated from European populations.
Mohammed et al. (73)	Study recruited 99 T1D and 206 controls. HLA-DRB1 genotyping was accomplished in 76 T1D and 198 controls. For genetic results only 56 T1D.	HLA-DRB1 alleles exhibited significant T1D association: three alleles (DRB1*03:01, DRB1*04:02, and DRB1*04:05) were positively associated, while three (DRB1*10:01, DRB1*15:02, and DRB1*15:03) were protective. DRB1*03:01 had the strongest association (OR = 5.04, p = 1.7x10^−10^).	HLA DR3/DR4 are associated with T1D. DRB1*15 seems to be protective for T1D. ^***^Limitation includes the small sample size.
Besancon et al. (63)	The study involved 132 individuals with young-onset diabetes (age < 21 years) in Mali. 100 individuals with young-onset diabetes and 200 controls undersent genotyping using NGS for the HLA-DRB1 exon 2. Genotype data available for 98 individuals with T1D and 195 controls	Some HLA-DRB1 alleles showed significant association with T1D compared to controls; DRB1*04:05, OR of 14.76 (95%CI 4.99-58.83), DRB1*09:01, OR of 3.48 (95%CI 1.52-8.33) and DRB1*03:01, OR of 2.82 (95%CI 1.59-5.03). DRB1*15:03 was protective for T1D with OR 0.27 (95%CI 0.09-0.65).	HLA DR3 and DR4 are associated with T1D. Results also show a significantly stronger association of the HLA-DRB1*04 allele compared to the DRB1*03 allele. ^****^However, study may be limited because of the small sample size given the wide CI for the DRB1*04 results.

HLA, Human leukocyte antigen; IDDM; Insulin-dependent diabetes mellitus; GADA, Glutamic acid decarboxylase autoantibody; GRS, Genetic risk score; GWAS, Genome-wide association studies; NGS, Next generation sequencing; PCR, Polymerase chain reaction; RFLP, Restriction fragment length polymorphism; SNP, Single nucleotide polymorphism; T1D, Type 1 diabetes. ^***^, ^****^was used to mean Take Note.

The HLA loci are highly polymorphic and vary significantly from one population to another (122). A combination of HLA DR3 and DR4 is known to confer the greatest genetic susceptibility to type 1 diabetes in Caucasian populations, while HLA-DR15 haplotypes are protective (87). The mechanisms underpinning the interaction between different HLA haplotypes to engender susceptibility or protection are poorly understood. Furthermore, very little is known about these interactions in African populations. The HLA DR4 haplotype has been demonstrated to be strongly associated with type 1 diabetes in Caucasian populations. Contrariwise, small-scale studies from African populations have shown a lower relative contribution of the HLA DR4 haplotype to type 1 diabetes susceptibility. The HLA DR3 type 1 diabetes susceptibility risk alleles are consistent with what has been observed in African Americans (11). Previous small-scale HLA genotyping studies in Cameroon and other African populations have pointed to the strong association between HLA DR3 compared to DR4 risk alleles and type 1 diabetes genetic susceptibility (119, 120). A recent study amongst newly diagnosed children and adolescents (age <21 years) and healthy controls from Mali showed that HLA DR4 risk alleles [OR 14.76 (95%CI 4.99–58.83)] were more strongly associated with T1D than HLA DR3 risk alleles [OR 2.82 (955CI 1.59–5.03)] (63). These findings generally confirm the association of HLA DR3 and DR4 haplotypes to T1D in this African population, though however limited by the small sample sizes of the groups analyzed. HLA DR3 allele-associated type 1 diabetes risk has also been described in Asian populations (123). Whether the differences in the HLA risk profiles between African and Caucasian populations contribute to the pattern and combinations of islet autoantibodies is unknown. Some studies have suggested that other HLA risk profiles, such as DR7 and DR9 genes, confer extremely high type 1 diabetes susceptibility in African-ancestry populations, although this has not been well elucidated (124). Nevertheless, the differences in HLA haplotypes observed in Africans and Caucasians with type 1 diabetes may further point to the distinct T1D endotypes, given the association between HLA haplotypes, islet autoantibody type, and immunohistological patterns that have recently been described. The hyperimmune T1D endotype (common in children diagnosed <7 years) is also referred to as T1DE1 or “proinsulin autoimmune-DR4” (PADR4) given that it is strongly associated with the HLA DR4 haplotype. Conversely, the pauci-immune T1D endotype (common in children diagnosed >13 years) is also called T1DE2 or the “GAD autoimmune-DR3” (GADR3) endotypes since it is strongly associated with HLA DR3 haplotype (70).

These endotypic immunogenetic differences may explain, in part at least, the reason why many African studies have reported a late-onset phenotype of T1D. There are suggestions that the rate of immune aggression may differ due to the pace of the breach to tolerance to pro(insulin) on the HLA DR4 haplotype compared to a slow-paced breach of tolerance to GAD peptides on the HLA DR3 haplotype (70, 73). However, observational and immunohistological studies from African populations will be needed to confirm this hypothesis.

### Use of genetic risk scores in African populations

No single gene is either necessary or sufficient to predict the development of the condition, and multiple genes contribute to genetic susceptibility. These findings have led to the development of type 1 diabetes genetic risk scores (T1DGRS) (125). These scores provide a measure of genetic susceptibility to T1D based on information from previous studies of genetic change associated with the disease risk. The scoring system is based on combining Single Nucleotide Polymorphisms (SNPs) in the HLA region and non-HLA loci (126). There is no T1DGRS specific to people with type 1 diabetes in sub-Saharan Africa. However, Balcha and colleagues studied a 19 SNP T1DGRS using SNPs initially developed from populations of European ancestry. They found that the GRS was markedly different between those with diabetes vs. control participants in their study and was substantially higher in autoantibody-positive diabetes cases than those with islet autoantibody-negative diabetes. This result suggests that T1DGRS developed using SNPs from European populations may still help discriminate T1D from other diabetes types in other populations. A similar finding has been reported by Harrison and colleagues, who applied a 9 SNP T1DGRS developed from populations of European origins to a group of people with T1D, T2D and controls in India (123). Although there are always concerns regarding the transferability of GRSs, these findings underscore the point that T1DGRSs developed using SNPs from populations of European ancestry may help discriminate between T1D and other diabetes types in other populations while pending population-specific genetic studies and development of population specific genetic risk scores.

### Limitations to type 1 diabetes genetic studies in sub-Saharan Africa

Most of these studies reporting the association of HLA genes and T1D in SSA are limited by their small sample size and type 1 diabetes case definition, primarily based on clinical features. Implications are that the frequency of the targeted genes and the effect size are lowered than expected for typical HLA genes known to contribute to T1D in other populations. This could explain why DR4 genes were not commonly reported in most studies. HLA genes are thought to be very stable across different ethnic groups and therefore are the main predicting factors of T1D genetic susceptibility (87). Also, the HLA genotyping methods employed were very dated compared to the powerful and robust contemporary genotyping methods available.

Few studies have examined the proportion of HLA risk alleles between those with islet autoantibody-positive and islet autoantibody-negative “non-autoimmune” diabetes. This is important to confirm whether participant with a clinician diagnosis of T1D and islet autoantibody-negative diabetes actually have antibody negative autoimmune T1D (suggested by genetic susceptibility HLA risk alleles) or suggested non-autoimmune disease in African populations. For example, Balcha and colleagues showed that the proportion of individuals with susceptible HLA DR3 (DRB1^*^03:01) haplotype was significantly higher in those with islet autoantibody-positive (52.9%) than those with islet autoantibody-negative diabetes [31.4% (61)]. However, the proportion DRB1^*^03:01 haplotype in those clinically diagnosed with T1D was 43.1% compared to 26.3% in controls. The reduction in the proportion of the individuals with susceptible HLA in the overall diabetes group may point to the presence of non-autoimmune diabetes especially amongst the group of individuals with islet autoantibody-negative diabetes. However, more detailed studies in other African settings are needed to confirm this hypothesis.

In summary, despite numerous methodological limitations, HLA DR3 genes have been reported in the few genetic studies conducted in SSA. Therefore, there is a need for extensive population-based studies (Genome-wise associated studies, GWAS) to be conducted in SSA to examine the relative contribution of the HLA genes, including the existing non-HLA loci known to contribute to T1D genetic susceptibility in other populations. Also, high throughput HLA genotyping studies (coupled with robust immunological methods) are needed to exclude the possible presence of a non-autoimmune form of diabetes common in children and young adults in African populations who present with T1D-like phenotype and who are negative for all commonly tested islet autoantibodies.

## Perspectives and research priorities for type 1 diabetes research in sub-Saharan Africa

Many questions remain unanswered concerning the true phenotype of type 1 diabetes in sub-Saharan Africa. Some of these questions are;

- Is type 1 diabetes different in sub-Saharan African populations when misclassification and effects of poor survival have been excluded?
- Is the late-onset insulin-dependent diabetes mellitus previously described in African populations an accurate description of a distinct clinical entity or due to selection bias caused by the survival of older individuals?
- If there is a late-onset phenotype, what might be the reasons for this decade-long age gap compared to the earlier onset of diabetes observed in Caucasian populations?
- Is preserved endogenous insulin secretion a reality in young-onset diabetes or a result of misclassification and/or survival bias, and what are the determining factors?
- What is the etiology of non-autoimmune type 1 diabetes phenotype in sub-Saharan Africa?

These questions are essential to shape type 1 diabetes research and care in the African continent. Therefore, we recommend that these questions and many more can be effectively tackled by creating centers of excellence in diabetes research in sub-Saharan Africa that can leverage the experience of long-standing international diabetes research institutions in a wide-and-fair manner. Transformational training and capacity-building of young and dynamic African Research Champions interested in focusing on type 1 diabetes research and care should be the bedrock of such initiatives to ensure sustainability. We suggest that these research priority areas should focus and not exclusively on:

- The creation of an active up-to-date research culture mixed with training and capacity building especially toward the set-up of local laboratories to carry out robust C-peptide and islet autoantibody testing in the African continent.
- The development of population-specific islet autoantibody positivity thresholds with appropriate standardization within the Diabetes Antibody Standardization Programme (DASP) workshop packages to better interpret the results of islet cell immunological studies.
- Factors that may be determinant in developing non-autoimmune young-onset diabetes in sub-Saharan Africa.
- The leverage of high-resolution HLA typing methods and GWAS in large dataset studies to better understand the genetic susceptibility of T1D in sub-Saharan African populations.
- Prospective cohort studies of well-defined T1D patients across multiple sites in SSA to study disease progression, treatment response and the incidence of long-term complications.
- The impact of childhood diabetes programmes (insulin donation schemes) on mortality associated with T1D in the African continent.

## Author contributions

All authors listed have made a substantial, direct, and intellectual contribution to the work and approved it for publication.
